# Health-related quality of life among military HIV patients on antiretroviral therapy

**DOI:** 10.1371/journal.pone.0178953

**Published:** 2017-06-07

**Authors:** Leonard Emuren, Seth Welles, Alison A. Evans, Marcia Polansky, Jason F. Okulicz, Grace Macalino, Brian K. Agan, the Infectious Disease Clinical Research Program HIV Working Group

**Affiliations:** 1 Department of Epidemiology and Biostatistics, Dornsife School of Public Health, Drexel University, Philadelphia, PA, United States of America; 2 Public Health Program, South University, Virginia Beach, VA, United States of America; 3 Infectious Disease Clinical Research Program, Department of Preventive Medicine and Biostatistics, Uniformed Services University of the Health Sciences, Bethesda, MD, United States of America; 4 Infectious Disease Service, San Antonio Military Medical Center, San Antonio, TX, United States of America; 5 Henry M. Jackson Foundation for the Advancement of Military Medicine, Inc., Bethesda, MD, United States of America; Imperial College London, UNITED KINGDOM

## Abstract

**Objective:**

The aims of this study were: (i) to determine the factors associated with HRQOL at baseline in our cohort, and (ii) to evaluate if there are differences in baseline HRQOL measures by antiretroviral treatment.

**Methods:**

The Short Form 36 (SF-36) was administered between 2006 and 2010 among members of the United States HIV Natural History Study cohort (NHS), and participants who completed the SF-36 were included in the study. Physical component summary (PCS) and mental component summary (MCS) scores were computed based on standard algorithms. Multivariate linear regression models were constructed for PCS and MCS to estimate the association between selected variables and HRQOL scores.

**Results:**

Antiretroviral therapy (ART) was not independently associated with HRQOL scores. Factors associated with PCS were CD4+ count < 200 cells/mm^3^ (β = -5.84, 95% CI: -7.63, -4.06), mental comorbidity (β = -2.82, 95% CI: -3.79, -1.85), medical comorbidity (β = -2.51, 95% CI: -3.75, -1.27), AIDS diagnosis (β = -2.38, 95% CI: -3.79, -0.98). Others were gender, military rank, marital status, and age. Factors independently associated with MCS were CD4+ count < 200 cells/mm^3^ (β = -1.93, 95% CI: -3.85, -0.02), mental comorbidity (β = -6.25, 95% CI: -7.25, -5.25), age (β = 0.37, 95% CI: 0.14, 0.60), and being African American (β = 1.55, 95% CI: 0.63, 2.47).

**Conclusion:**

Among military active duty and beneficiaries with HIV, modifiable factors associated with HRQOL measures included advanced HIV disease, and mental or medical comorbidity. Addressing these factors may improve quality of life of HIV-infected individuals in the NHS cohort.

## Introduction and background

The annual estimated rate of new human immunodeficiency virus (HIV) infections in the United States between 2008 and 2011 remained stable at 15.8 per 100,000 while the rate for HIV stage 3 or acquired immune deficiency syndrome (AIDS) was 10.3 per 100,000 during the same period[1]. Death from HIV/AIDS has continued to decline since the mid-1990s with the introduction of highly active antiretroviral therapy (HAART)[2, 3]. By 2010, the Centers for Disease Control and Prevention (CDC) estimated that the all-cause mortality in people infected with HIV in the United States was 6.3 per 100,000 and the all-cause mortality in those with a diagnosis of AIDS was 5.0 per 100,000[1]. Given the stable incidence of HIV/AIDS in the US and the declining mortality among infected individuals, greater emphasis is now being placed on other end-point measures both in clinical and public health settings, such as health-related quality of life (HRQOL), in assessing the well-being of individuals living with HIV/AIDS[4, 5].

HRQOL is a multidimensional and dynamic concept that is well recognized as an end-point in assessing the well-being of individuals living with HIV/AIDS[5–9]. Several factors have been established as determinants of HRQOL in HIV-infected populations but these determinants are partly influenced by the population studied, the HRQOL instrument used and the country of study among other factors[10, 11]. Some determinants of HRQOL in HIV-infected individuals in the United States and other high-income countries[12] are age[13, 14], race/ethnicity[13], gender[7, 8, 12, 15], educational level[13], income level[13, 14], socioeconomic status[16], access to health insurance[17], being on antiretroviral therapy[9, 10], injection drug use[18], the presence of mental and medical comorbidities[14, 19], presence of AIDS-defining illnesses[13, 20], CD4+ cell count[13, 21], plasma viral load (pVL)[21], and less frequently captured variables such as coping style/ability[17, 22, 23] and social support[22] among others. Marital status has also been shown to be associated with HRQOL in a large representative sample of the U.S. military[24].

The relationship between HIV/AIDS, HAART and HRQOL is complex. While HAART helps to prevent disease progression and results in better quality of life and well-being in HIV-infected individuals, the prolonged use of medication that is necessary to continually keep viral suppression below detection levels may lead to adverse effects that may reduce an individual’s HRQOL. Such side effects of HAART, including lipodystrophy, diarrhea and other medication-related symptoms, have also been shown to affect HRQOL[25–27]. Although, side effects are not specific to one class of HAART medications, protease inhibitors have been implicated as having greater adverse effects including morphological changes and metabolic disturbances[28]. However, most studies evaluating the impact of different HAART regimen on HRQOL have been in clinical trials[10, 29–32] or following a switch from protease inhibitor-based regimens to non-protease-inhibitor regimens without the benefit of an appropriate control group[28].

Some predictors of HRQOL in HIV-infected individuals in the general US population, such as lack of access to healthcare due to lack of insurance, access to and maintenance of antiretroviral medications, and injection drug use may not play an equally important role as determinants of HRQOL of HIV-infected individuals in the United States Military. HRQOL has not been previously evaluated in the U.S. Military HIV Natural History Study (NHS), which is one of the oldest open-enrollment dynamic HIV cohorts in the country and provides a unique opportunity to evaluate HRQOL in the setting of equal access to healthcare. Furthermore, racial diversity and equal access to medication are other advantages of our cohort. Also, previous study in the military shows that injection drug use is rare among military personnel[33, 34]. The aims of this study were therefore: (i) to determine the factors associated with HRQOL at baseline in the U.S. military cohort, and (ii) to evaluate if there are differences in HRQOL scores by treatment group.

## Methods

The U.S. Military HIV Natural History Study has been approved centrally by the Uniformed Services University Institutional Review Board (IRB) and at each participating site and is conducted according to the principles expressed in the Declaration of Helsinki. Written informed consent was obtained from the participants. This analysis was approved by the central IRB and Drexel University.

### Study cohort

The NHS is a prospective multicenter continuous enrollment observational cohort of HIV-infected active duty military personnel and other beneficiaries (spouses, adult dependents, and retired military personnel) from the Army, Navy/Marines and Air Force enrolled since 1986[33, 35–37]. Participants are followed at six medical centers in the United States. Demographic data are collected at baseline and updated while medical and medication histories and standard laboratory studies are collected biannually. Blood samples obtained from participants in this cohort from scheduled visits are stored in a repository. All NHS participants provided informed consent, and approval for this research was obtained from the institutional review board at each participating site.

### Study participants

The RAND Short Form 36 (SF-36) questionnaires were administered annually to NHS participants from April 2006 to September 2010. However, a few participants had more than one completed questionnaire in a year, and for these participants the last completed questionnaire for that year was used. Baseline was defined as the first ever HRQOL measure irrespective of when the participant was first enrolled in the NHS.

### Definitions and variable selections

Variable selection was based on the literature on HRQOL in HIV-infected individuals in the United States and other high income countries[5, 10], on HRQOL in the US Military[24] and on variables captured in our cohort[33–36].

#### Health-related quality of life scores

We computed the norm-based physical (PCS) and mental (MCS) component summary scores from the eight health domains in the Short Form 36 (SF-36) questionnaire in line with the recommended scoring algorithm for the RAND 36-item health survey 1.0[38, 39]. The PCS and MCS scores were the outcome variables in our analyses. We have reported only the summary scores here for ease of interpretation of results and for comparison with other studies.

#### HAART definition

HAART was defined as a combination of at least three full dose antiretroviral agents similar to previous investigations for this cohort[33]. HAART treatment was the main explanatory variable. HAART was divided into four groups: protease inhibitor-based HAART (PI-HAART), for HAART with at least one protease inhibitor in the HAART regimen; non-protease-inhibitor-based HAART (NPI-HAART), for HAART with no protease inhibitor in the HAART regimen; HAART-naïve group (HAART-N) for those who had never been on HAART prior to completing the HRQOL survey; and, OFF-HAART group made up of participants who were not on HAART at the time of completing the survey but had prior use of HAART.

#### Covariates

Covariates considered for inclusion in our models were based on previous studies as well as on the demographic and clinical characteristics that were captured in the NHS cohort. These covariates included gender (male/female), age, military rank (officer/warrant officer, enlisted and civilian/retired), marital status (married, not married), race/ethnicity (non-Hispanic white, non-Hispanic African-American, and others), pVL (>50 copies/mL or ≤50 copies/mL), CD4+ cell count (<200 cells/mm^3^, 200–499 cells/mm^3^ and >499 cells/mm^3^), medical comorbidity, mental comorbidity, AIDS-defining illnesses (1993 CDC criteria), HIV duration, and calendar year. We used the CD4+ cell count and pVL values closest in time to the HRQOL measure used. Although most of the participants were not new to the NHS, administration of the HRQOL questionnaire began in 2006 and continued until 2010. We therefore included calendar year to adjust for any temporal variations in participants’ characteristics upon entry into the HRQOL study. Medical comorbidity referred to concurrent chronic medical conditions such as diabetes mellitus, hypertension and cancers the participants had at the time of the study. Similarly, mental comorbidity included such conditions as major depressive disorder, general anxiety disorder, bipolar disorder and alcohol abuse. Both medical and mental comorbidities were extracted from the participants’ medical record using the central electronic health-records system of the US Military. Medical comorbidity was classified as having “no” for participants who had no medical comorbidity or “yes” for those with one or more medical comorbidity. Mental comorbidity was similarly classified.

### Inclusion and exclusion criteria

All participants aged 18 years and above who completed the HRQOL survey questionnaires between 2006 and 2010 were eligible for the study. We excluded participants who had been on treatment for less than four weeks prior to taking the HRQOL survey since some of the questions in the questionnaire specifically asked for participants’ functional health in the past four weeks. We further excluded participants who were on both on PI-HAART and NPI-HAART within four weeks of taking the survey Finally, we excluded participants who were on a non-HAART antiretroviral therapy at the time of survey.

### Statistical analyses

We summarized the baseline characteristics of the participants who met our inclusion criteria by four HAART groups. Proportions of participant’s characteristics were compared using Chi-square tests while the medians of the numeric variables were compared using the Kruskal-Wallis tests. Separate multivariate regression models were constructed for PCS and MCS scores. We tested the effect of covariates on participants’ PCS and MCS scores in univariate analyses, and included those which achieved a significance p-value of less than 0.05 in the final multivariate analyses. We also checked for evidence of interaction between HAART (the treatment variable), and covariates. Finally, we checked for any evidence of multi-collinearity for all covariates using the variance inflation factor (VIF). All statistical analyses were performed using SAS 9.3 [SAS Institute Inc., Cary, NC].

## Results

**Fig 1:**
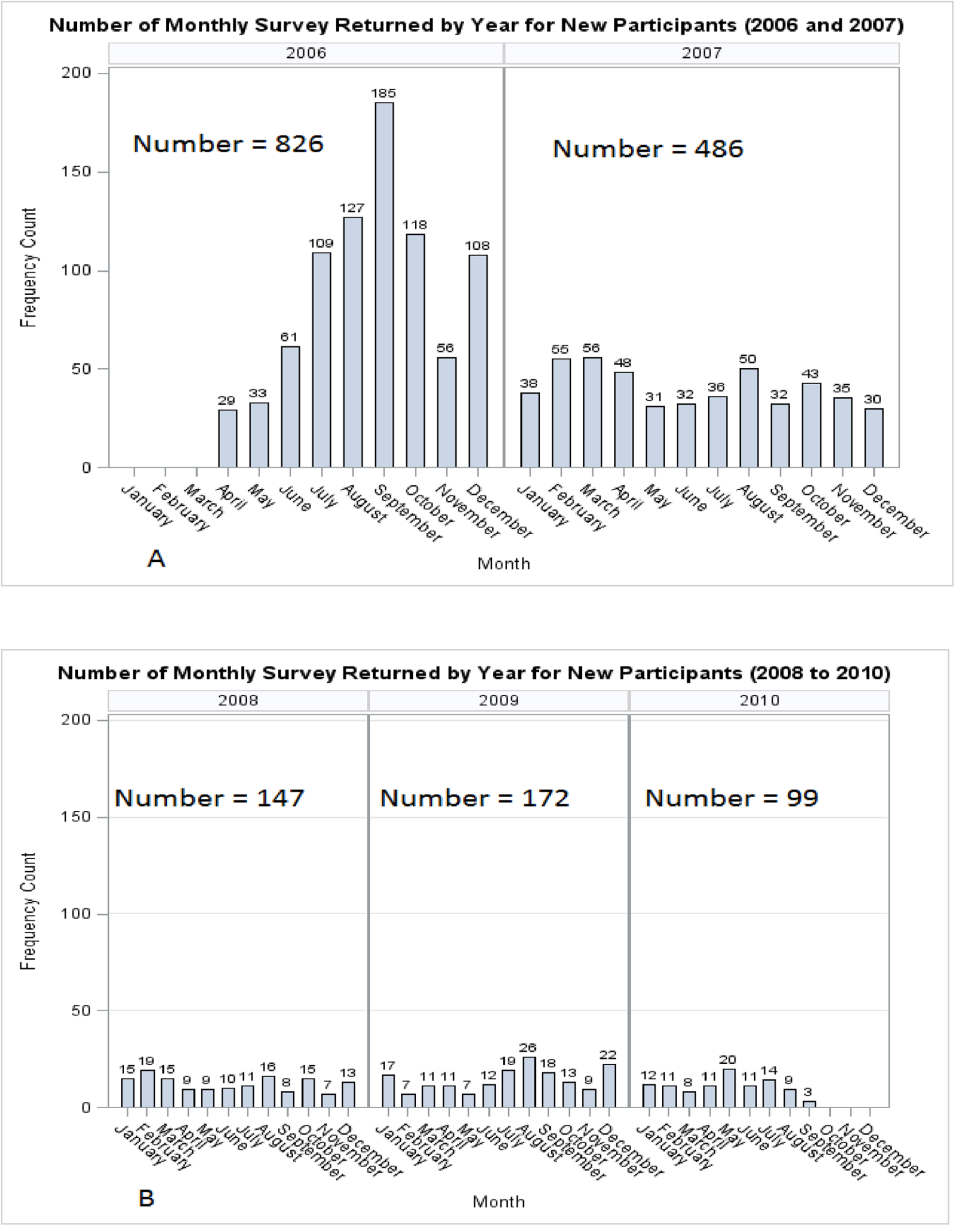
HRQOL survey return by month and calendar year.

Baseline demographic and clinical characteristics by HAART group for participants with SF-36 data are displayed in Tables 1 and 2. Fig 1A and 1B show participants monthly returns of survey questionnaires from 2006 to 2010. Of the 1730 eligible participants, 24 (1.4%) on a non-HAART antiretroviral therapy were excluded. We also excluded another 38 (2.2%) who were either on HAART for less than 4 weeks prior to the survey or on both PI/NPI-HAART within 4 weeks of survey completion. Demographic characteristics with the exception of gender and marital status varied by HAART group; participants were also different on their clinical characteristics, HIV disease indicators and HRQOL measures (Tables 1 & 2). There were, however, more dramatic differences in participants who were HAART naïve compared to the other HAART groups. They were younger, more likely to be other races, less likely to be retired, had a shorter duration of HIV infection, and more likely to have higher plasma viral load copies.

**Table 1:** Baseline demographic/background characteristics.

Characteristics	Total N = 1668 (%)	PI-Based HAART N = 519 (%)	Non-PI-Based HAART N = 582 (%)	HAART-Naïve N = 411 (%)	Off-HAART N = 156 (%)	P-Value*
Age (years), Median	**40.0**	**44.0**	**41.0**	**29.0**	**40.5**	**< .0001**
• IQR	**32.0–47.0**	**39.0–50.0**	**35.0–47.0**	**25.0–38.0**	**36.0–45.0**	
Gender						0.23
• Male	1552 (93.0)	485 (93.4)	533 (91.6)	390 (94.9)	144 (92.3)	
• Female	116 (7.0)	34 (6.6)	49 (8.4)	21 (5.1)	12 (7.7)	
Race/Ethnicity						**< .0001**
• Non-Hispanic White	**701 (42.0)**	**234 (45.1)**	**253 (43.5)**	**145 (35.3)**	**69 (44.2)**	
• Non-Hispanic African American	**703 (41.2)**	**223 (43.0)**	**251 (43.1)**	**164 (39.9)**	**65 (41.7)**	
• Hispanic/Others	**264 (15.8)**	**62 (11.9)**	**78 (13.4)**	**102 (24.8)**	**22 (14.1)**	
Rank						**< .0001**
• Officer/Warrant Officer	**121 (7.3)**	**25 (4.8)**	**44 (7.6)**	**41 (10.0)**	**11 (7.1)**	
• Enlisted	**883 (53.0)**	**177 (34.2)**	**290 (49.8)**	**340 (82.9)**	**76 (48.7)**	
• Civilian/Retired	**662 (39.7)**	**316 (61.0)**	**248 (42.6)**	**29 (7.1)**	**69 (44.2)**	
Marriage						0.18
• Yes	533 (32.0)	165 (31.8)	201 (34.5)	115 (28.0)	52 (33.3)	
• No	1135 (68.0)	354 (68.2)	381 (65.5)	296 (72.0)	104 (66.7)	
Calendar Year (of Enrollment)						**< .0001**
• Baseline Year 2006	**805 (48.3)**	**286 (55.1)**	**313 (56.1)**	**106 (25.8)**	**100 (64.1)**	
• Baseline Year 2007	**467 (28.0)**	**168 (32.4)**	**157 (31.7)**	**107 (26.0)**	**35 (22.4)**	
• Baseline Year 2008	**142 (8.5)**	**33 (6.4)**	**34 (5.8)**	**67 (16.3)**	**8 (5.1)**	
• Baseline Year 2009	**161 (9.7)**	**24 (4.6)**	**48 (8.2)**	**79 (19.2)**	**10 (6.4)**	
• Baseline Year 2010	**93 (5.6)**	**8 (1.5)**	**30 (5.2)**	**52 (12.7)**	**3 (1.9)**	
Physical Component Summary Score						**< .0001**
• Median	**55.0**	**52.9**	**55.4**	**56.3**	**53.0**	
• IQR	**47.2–58.0**	**43.9–57.2**	**48.1–58.2**	**51.3–58.7**	**45.2–57.4**	
Mental Component Summary Score						**0.0003**
• Median	**50.4**	**50.4**	**51.3**	**48.7**	**50.1**	
• IQR	**43.5–53.9**	**42.4–54.1**	**44.9–54.4**	**43.4–53.3**	**42.3–52.9**	

**Table 2:** Baseline HIV disease indicators and clinical data.

Characteristics	Total N = 1668 (%)	PI-Based HAART N = 519 (%)	Non-PI-Based HAART N = 582 (%)	HAART-Naïve N = 411 (%)	Off-HAART N = 156 (%)	P-Value*
AIDS						**< .0001**
• Yes	**192 (11.5)**	**123 (23.7)**	**55 (9.4)**	**2 (0.5)**	**12 (7.7)**	
• No	**1476 (88.5)**	**396 (76.3)**	**527 (90.6)**	**409 (99.5)**	**144 (92.3)**	
Duration of HIV infection (years)						**< .0001**
• Median	**8.0**	**15.0**	**8.0**	**0.0**	**11.0**	
• IQR	**2.0–15.0**	**10.0–19.0**	**4.0–14.0**	**0–1.0**	**7.0–15.0**	
CD4+ Cell Count (x 10^6^/L)						**< .0001**
• Median	**505.0**	**477.0**	**570.0**	**466.0**	**450.0**	
• IQR	**370.0–682.0**	**316.0–678.0**	**35.0–776.0**	**374.0–606.0**	**338.0–622.0**	
CD4+ Groups						**< .0001**
• CD4+ Less Than 200	**102 (6.1)**	**62 (12.0)**	**18 (3.1)**	**8 (2.0)**	**14 (9.0)**	
• CD4+ Between 200 and 499	**713 (42.8)**	**213 (41.0)**	**192 (33.0)**	**227 (55.4)**	**81 (51.9)**	
• CD4+ Greater Than 499	**851 (51.1)**	**244 (47.0)**	**371 (63.9)**	**175 (42.7)**	**61 (39.1)**	
Plasma Viral Load (Log_10_)						**< .0001**
• Median	**1.7**	**1.7**	**1.7**	**4.3**	**4.0**	
• IQR	**1.7–4.0**	**1.7–2.3**	**1.7–1.7**	**3.7–4.7**	**3.3–4.5**	
Plasma Viral Load > 50 copies/mL						**< .0001**
• Yes	**823 (49.4)**	**176 (34.0)**	**103 (17.7)**	**405 (98.5)**	**139 (89.1)**	
• No	**844 (50.6)**	**342 (66.0)**	**479 (82.3)**	**6 (1.5)**	**17 (10.9)**	
Mental Comorbidity						**< .0001**
• Yes	**430 (25.8)**	**190 (36.6)**	**150 (25.8)**	**36 (8.8)**	**54 (34.6)**	
• No	**1238 (74.2)**	**329 (63.4)**	**432 (74.2)**	**375 (91.2)**	**102 (65.4)**	
Medical Comorbidity						**< .0001**
• Yes	**240 (14.4)**	**126 (24.3)**	**84 (14.4)**	**6 (1.5)**	**24 (15.4)**	
• No	**1428 (85.6)**	**393 (75.7)**	**498 (85.6)**	**405 (98.5)**	**132 (84.6)**	

Table 3 shows the unadjusted and adjusted analyses for the PCS scores. Factors associated with lower PCS scores were age, CD4+ cell count <200 cells/mm^3^, lower military rank or being civilian/retired, presence of medical and mental comorbidities, AIDS diagnosis, and being married. Being male was associated with a higher PCS score. Also, participants who were administered the HRQOL questionnaire in 2007 had a higher PCS score compared to their 2006 counterparts but there were no statistically significant differences in PCS scores for participants enrolled in 2008 to 2010 compared to their 2006 counterparts. The PCS scores of participants receiving protease inhibitors were significantly lower than those receiving non-protease inhibitors in the unadjusted model (β = -2.59, 95% Confidence Limits [95% CL]: -3.64, -1.55) but not in the adjusted model. The PCS scores of participants in the Off-HAART group were also significantly lower than those receiving non-protease inhibitors in the unadjusted model (β = -1.79, 95% CL: -3.35, -0.25) but not in the adjusted model (β = -1.11, 95% CL: -2.61, 0.38). Compared to participants receiving non-protease inhibitors, HAART-naivety was associated with a higher PCS scores by 1.99 (95% CL: 0.88, 3.11) in the unadjusted model but not in the adjusted model. Similarly, PCS scores in the PI-HAART group was significantly different from those of in the Off-HAART group and the HAART-naïve in the unadjusted model but not after adjustment (see Table 4). There were no statistically significant differences in PCS scores by pVL category and HIV duration in the adjusted model.

**Table 3:** Factors associated with physical component summary scores at baseline.

Variable	Physical Component Summary Scores							
	Unadjusted Model				Adjusted Model (n = 1652)			
	β Coefficient	SE	95% CI	p-Value	β Coefficient	SE	95% CI	p-Value
HAART Status*								
• HAART Naïve	**1.99**	**0.57**	**0.88, 3.11**	**0.0005**	-0.52	0.63	-1.76, 0.72	0.41
• Off-HAART	**-1.79**	**0.80**	**-3.35, -0.23**	**0.02**	-1.12	0.76	-2.61, 0.38	0.14
• PI-Based HAART	**-2.59**	**0.53**	**-3.64, -1.55**	**< .0001**	-0.71	0.54	-1.77, 0.35	0.19
• Non-PI-Based HAART	**-**	**-**	**-**	**-**	-	-	-	-
Age (Years, 5-yearly Increment)	**-1.02**	**0.10**	**-1.22, -0.82**	**< .0001**	**-0.50**	**0.14**	**-0.77, -0.23**	**0.0003**
Gender								
• Male	**2.99**	**0.86**	**1.30, 4.68**	**0.0005**	**2.27**	**0.83**	**0.64, 3.90**	**0.006**
• Female	**-**	**-**	**-**	**-**	**-**	**-**	**-**	**-**
Race/Ethnicity								
• Non-Hispanic African American	0.07	0.48	-0.88, 1.01	0.89				
• Hispanic/Others	0. 33	0.65	-0.95, 1.61	0.61				
• Non-Hispanic White	-	-	-	-				
Rank								
• Enlisted	**-2.02**	**0.84**	**-3.68, -0.36**	**0.02**	**-1.94**	**0.83**	**-3.56, -0.32**	**0.02**
• Civilian	**-6.11**	**0.86**	**-7.80, -4.41**	**< .0001**	**-3.23**	**0.88**	**-4.96, -1.50**	**0.0003**
• Officer/Warrant Officer	**-**	**-**	**-**	**-**	-	-	-	-
Marital Status								
• Married	**-1.75**	**0.47**	**-2.67, -0.82**	**0.0002**	**-1.23**	**0.45**	**-2.11, -0.35**	**0.006**
• Single	**-**	**-**	**-**	**-**	-	-	-	-
CD4+ Cell Count Groups								
• Less Than 200	**-7.75**	**0.93**	**-9.57, -5.93**	**< .0001**	**-5.84**	**0.91**	**-7.63, -4.06**	**< .0001**
• Between 200 and 499	-0.59	0.45	-1.47, 0.29	0.19	-0.70	0.43	-1.55, 0.15	0.11
• Greater than 499	-	-	-	-	**-**	**-**	**-**	**-**
Plasma Viral Load >50 copies/mL								
• Yes	0.57	0.44	-0.30, 1.43	0.20				
• No	-	-	-	-				
Medical Comorbidity								
• Yes	**-5.08**	**0.62**	**-6.29, -3.87**	**< .0001**	**-2.51**	**0.63**	**-3.75, -1.27**	**< .0001**
• No	**-**	**-**	**-**	**-**	-	-	-	-
Mental Comorbidity								
• Yes	**-4.60**	**0.49**	**-5.56, -3.63**	**< .0001**	**-2.82**	**0.49**	**-3.79, -1.85**	**< .0001**
• No	**-**	**-**	**-**	**-**	-	-	-	-
AIDS								
• Yes	**-6.09**	**0.68**	**-7.42, -4.76**	**< .0001**	**-2.38**	**0.72**	**-3.79, -0.98**	**0.0009**
• No	**-**	**-**	**-**	**-**	-	-	-	-
Duration of HIV infection (per 5 years)	**-1.49**	**0.15**	**-1.79, -1.20**	**< .0001**	0.05	0.23	-0.41, 0.51	0.83
Calendar Year								
• 2010	1.87	0.98	-0.06, 3.79	0.06	-0.52	0.95	-2.38, 1.35	0.59
• 2009	0.74	0.77	-0.78, 2.25	0.34	-0.46	0.75	-1.92, 1.01	0.54
• 2008	**2.58**	**0.82**	**0.97, 4.19**	**0.002**	1.38	0.78	-0.16, 2.92	0.08
• 2007	**1.05**	**0.52**	**0.03, 2.08**	**0.04**	**1.47**	**0.49**	**0.51, 2.43**	**0.003**
• 2006	-	-	-	-	-	-	-	-
***Intercept***	***NA***	***NA***	***NA***	***NA***	**58.14**	**1.62**	**54.97, 61.31**	**< .0001**

*F statistics for univariate HAART status is 22.28 with a corresponding p-value of < .0001

**Table 4:** Unadjusted and adjusted MCS scores for HAART with PI-HAART as the referent category.

Variable	Physical Component Summary Scores							
	PCSS Unadjusted Model				PCSS Adjusted Model^$^ (n = 1652)			
	β Coefficient	SE	95% CI	p-Value	β Coefficient	SE	95% CI	p-Value
HAART Status**								
• HAART Naïve	**4.59**	**0.58**	**3.44, 5.73**	**< .0001**	0.19	0.74	-1.25, 1.64	0.79
• Off-HAART	0.81	0.81	-0.77, 2.39	0.32	-0.40	0.77	-1.92, 1.11	0.60
• Non-PI-Based HAART	**2.59**	**0.53**	**1.55, 3.64**	**< .0001**	0.71	0.54	-0.35, 1.77	0.19
• PI-Based HAART	**-**	**-**	**-**	**-**	-	-	-	-

**F statistics for univariate HAART status is 22.28 with a corresponding p-value of < .0001 same as for Table 3 above

^$^Adjusted for same variables as in Table 3

Table 5 shows the unadjusted and adjusted analyses for the MCS scores. Increasing age and being African American were associated with relatively higher MCS scores. Factors independently associated with lower MCS scores were CD4+ cell count <200 cells/mm^3^ (β = -1.93, 95% CL: -3.35, -0.05) and mental comorbidity (β = -6.25, 95% CL: -7.25, -5.25). Plasma viral load >50 copies/mL and AIDS diagnosis were associated with lower MCS scores in the unadjusted models but not in the adjusted model. The MCS scores of participants receiving protease inhibitors were not significantly different from those of receiving non-protease inhibitors. MCS scores were 1.44 and 2.34 significantly lower for the HAART-Naïve and Off-HAART groups respectively compared to those on NPI-HAART. These differences were no longer significant after adjustment. On the other hand, the PI-HAART group did not show any differences in MCS scores before and after adjustment with the HAART-naïve and Off-HAART groups (Table 6). We did not find any evidence of multi-collinearity or significant interaction in the final multivariate models.

**Table 5:** Factors Associated with mental component summary scores at baseline.

Variable	Mental Component Summary Scores							
	Unadjusted Model				Adjusted Model (n = 1654)			
	β Coefficient	SE	95% CI	p-Value	β Coefficient	SE	95% CI	p-Value
HAART Status*								
• HAART Naïve	-1.44	0.59	-2.60, -0.29	0.01	-1.20	0.78	-2.73, 0.33	0.12
• Off-HAART	-2.34	0.82	-3.96, -0.73	0.004	-1.13	0.89	-2.87, 0.61	0.20
• PI-Based HAART	-0.94	0.55	-2.02, 0.15	0.09	-0.07	0.55	-1.14, 1.01	0.90
• Non-PI-Based HAART	-	-	-	-	-	-	-	-
Age (Years, 5-yearly Increment)	**0.25**	**0.11**	**0.04, 0.46**	**0.02**	**0.37**	**0.12**	**0.14, 0.60**	**0.002**
Gender								
• Male	0.84	0.88	-0.89, 2.57	0.34				
• Female	-	-	-	-				
Race/Ethnicity								
• Non-Hispanic African American	**1.84**	**0.49**	**0.88, 2.79**	**0.0002**	**1.55**	**0.47**	**0.63, 2.47**	**0.001**
• Hispanic/Others	-0.81	0.66	-2.10, 0.48	0.23	-0.74	0.64	-2.00, 0.51	0.24
• Non-Hispanic White	-	-	-	-	-	-	-	-
Rank								
• Enlisted	-0.36	0.88	-2.08, 1.37	0.68				
• Civilian	-1.19	0.90	-2.95, 0.57	0.18				
• Officer/Warrant Officer	-	-	-	-				
Marital Status								
• Married	-0.31	0.48	-1.26, 0.63	0.52				
• Single	-	-	-	-				
CD4+ Cell Count Groups								
• Less Than 200	**-3.07**	**0.96**	**-4.95, -1.19**	**0.001**	**-1.93**	**0.98**	**-3.85, -0.02**	**<0.05**
• Between 200 and 499	**-1.04**	**0.46**	**-1.95, -0.13**	**0.02**	-0.75	0.46	-1.65, 0.15	0.10
• Greater than 499	**-**	**-**	**-**	**-**	**-**	**-**	**-**	**-**
Plasma Viral Load >50 copies/mL								
• Yes	**-1.46**	**0.45**	**-2.34, -0.58**	**0.001**	-0.41	0.61	-1.60, 0.79	0.51
• No	**-**	**-**	**-**	**-**	-	-	-	-
Medical Comorbidity								
• Yes	0.71	0.64	-0.54, 1.97	0.26				
• No	-	-	-	-				
Mental Comorbidity								
• Yes	**-5.99**	**0.49**	**-6.96, -5.03**	**< .0001**	**-6.25**	**0.51**	**-7.25, -5.25**	**< .0001**
• No	**-**	**-**	**-**	**-**	-	-	-	-
AIDS								
• Yes	**-1.97**	**0.71**	**-3.36, -0.59**	**0.005**	-0.88	0.73	-2.31, 0.55	0.23
• No	**-**	**-**	**-**	**-**	-	-	-	-
Duration of HIV infection (per 5 years)	0.003	0.03	-0.06, 0.07	0.91				
Calendar Year								
• 2010	0.59	1.00	-1.37, 2.56	0.55				
• 2009	-0.28	0.79	-1.73, 1.34	0.72				
• 2008	-0.30	0.84	-1.83, 1.34	0.72				
• 2007	-0.45	0.53	-1.49, 0.60	0.40				
• 2006	-	-	-	-				
***Intercept***	***NA***	***NA***	***NA***	***NA***	***46*.*91***	***1*.*13***	***44*.*70*, *49*.*12***	***<* .*0001***

*F statistics for univariate HAART status is 3.66 with a corresponding p-value of 0.01

**Table 6:** Unadjusted and adjusted MCS scores for HAART with PI-HAART as the referent category.

Variable	Mental Component Summary Scores							
	MCSS Unadjusted Model				MCSS Adjusted Model^&^ (n = 1654)			
	β Coefficient	SE	95% CI	p-Value	β Coefficient	SE	95% CI	p-Value
HAART Status***								
• HAART Naïve	-0.51	0.61	-0.15, 2.02	0.40	-1.13	0.79	-2.68, 0.42	0.15
• Off-HAART	-1.41	0.83	-3.04, 0.23	0.09	-1.07	0.87	-2.78, 0.64	0.22
• Non-PI-Based HAART	0.94	0.55	-0.15, 0.68	0.09	0.07	0.55	-1.01, 1.14	0.90
• PI-Based HAART	-	-	-	*-*	-	-	-	-

***F statistics for univariate HAART status is 3.66 with a corresponding p-value of 0.01 same as for Table 5 above

^&^Adjusted for same variables as in Table 5

## Discussion

Factors independently associated with PCS scores in our cohort were age, CD4+ cell count <200 cells/mm^3^, mental comorbidity, military rank, marital status, gender, medical comorbidity, AIDS diagnosis, and baseline enrollment year being 2007. Age has been reported in the literature to be negatively associated with PCS score in HIV-infected populations[14, 15, 18, 40–42]. Also, Smith et al found age to be negatively associated with PCS in a non-HIV military population[24] which is consistent with our findings. The relationship between aging and HIV is complex, and how aging impacts physical functional health may be both indirect and direct. For example, both increasing age and HIV infection lead to gradual decline in immunity that may result in lower PCS scores. Furthermore, older individuals have slower immune recovery and achieve less CD4+ cell restoration with HAART[43] which may negatively impact PCS. Also, both HIV infection and aging are associated with increased medical comorbidities that could negatively impact PCS[19]. Beyond that, physical senescence associated with older age may also contribute to poorer PCS[5].

Akin to the literature, we found that CD4+ cell count <200 cells/mm^3^ was significantly associated with lower PCS score[13, 21, 44]. There was no significant difference in PCS scores of participants with CD4+ cell count of 200–499 cells/mm^3^ when compared to those with CD4+ cell count >499 cells/mm^3^, similar to findings by others[13, 14]. The negative impact of CD4+ cell count <200 cells/mm^3^ on PCS is likely attributable to the greater burden of the disease associated with CD4+ cell counts <200 cells/mm^3^, including the fact these individuals are more likely to have had HIV-infection for a longer period, be older and may have more associated comorbidities as was the case in our cohort (data not shown). Plasma viral load was, however, not associated with PCS similar to findings by others[14, 45, 46]. This is not entirely surprising since the effect of pVL on HRQOL may be partly explained by its effect on CD4+ cell count, and as previously noted by other investigators, CD4+ cell count is a better prognostic marker for disease progression for HIV-infected individuals on HAART[45, 46]. And in the NHS quality of life study, over 74% of the participants on HAART (Table 2) had pVL ≤50 copies/mL, a level that reflects the goal of therapy in suppressing viral activity.

The presence of medical and mental comorbidities was negatively associated with physical functional health similar to findings by others[7, 14, 19, 20, 22, 40]. Although diverse psychological comorbidities have been shown to influence HRQOL, depression, which accounted for over 60% of the psychological comorbidity in our cohort, is by far the most predictive of HRQOL[7, 14, 40]. Having ever been diagnosed with AIDS was negatively associated with PCS in our cohort similar to findings by others[13, 40, 47]. The median time since their last AIDS diagnosis in our cohort was 8 years (IQR: 2–12 years), and only 12 participants (6.12% of all those with AIDS at baseline) had a recent AIDS diagnosis in the one year preceding enrollment into the study. In sensitivity analyses, we did not find any differences in the results when we excluded these participants with a recent AIDS diagnosis.

We found that being male was significantly associated with a 2.2 point higher PCS score compared to being female in our cohort, a finding similar to what has been reported in the literature[7, 8, 12, 15, 48] including the US Military[24]. Summary score levels of 2 to 3 are considered clinically and socially relevant[24, 49], and it has been suggested that women are more likely to report their poor physical state than men because society expects men to adopt a more enduring attitude[5, 12, 50]. Also, women with HIV may face a number of gender inequalities that may exacerbate clinical disease[48], although it is doubtful if this may apply to the NHS cohort. Being civilian was associated with over a 3 point lower PCS score compared to being an officer which is not entirely surprising as physical fitness is a requisite condition for remaining in the military. However, it is less clear why those enlisted also have lower PCS scores compared to officers.

Being married was associated with lower PCS score in our cohort, a finding similar to that reported by Smith et al for a non-HIV military cohort[24]. Although we do not have additional data to support the association between marital status and PCS score, it is possible that being married makes participants more conscious of their physical limitations and increase their tendency to reporting those as captured in the HRQOL questionnaire. Also, those enrolled in the study in 2007 had a slightly higher PCS score compared to the 2006 enrollees. The SF-36 instrument was routinely administered from April 2006 to September 2010 (see Fig 1A and 1B). The greater majority of those who completed the questionnaire in 2006 and 2007 were already in the cohort while most those who completed the survey from 2008 onwards were new to the cohort. Because enrollment took place over a prolonged period (over four years), it was important to adjust for time (calendar year) to account for any temporal variations in enrollment, and not necessarily because Calendar Year by itself would be associated with quality of life. It is plausible that there may be differences between completing the questionnaire soon after entry into the study (a surrogate for recent HIV infection) and doing so after a few years. In our study, however, the difference in PCS scores was between 2006 and 2007, which could be an incidental finding.

Our findings indicate that there were statistically significant differences in PCS scores among HAART treatment groups in the unadjusted models (Tables 3 and 4) but not after adjustment for covariates. The differences in the unadjusted models may therefore be explained by the participants’ demographic and clinical indicators similar to the findings reported by Armon et al[17] and Preau et al[40]. Although HIV duration was negatively associated with perceived physical health in the unadjusted model, the association was no longer significant after adjusting for age and other covariates. Furthermore, age is often correlated with HIV duration as was in our cohort (correlation coefficient 0.62, p < .0001). Race/ethnicity was not associated with PCS in our cohort, which may give credence to the view that with employment, and/or equal access to healthcare, race/ethnicity is not significantly associated with PCS.

Factors independently associated with MCS scores in our cohort were age, CD4+ cell count <200 cells/mm^3^, mental comorbidity, and race/ethnicity. We found a positive association between increasing age and MCS in our cohort similar to that in the military[24] and in HIV-infected individuals[13]. The relationship between age and MCS is however not consistent[51], making others to conclude that mental health is less dependent on age[5]. We also found that CD4+ cell count <200 cells/mm^3^ was independently associated with lower MCS score similar to the findings by others[8, 17, 52] but unlike the findings by Hays et al[13], which found a positive association between lower CD4+ cell count and MCS scores. It has been suggested that because CD4+ cell count <200 cells/mm^3^ is associated with faster disease progression in HIV-infected individuals, this will tend to cause distress that may negatively impact MCS[8]. There was no significant association between pVL >50 copies/mL and MCS scores in the adjusted model, a finding that is similar to what others have reported[14, 45, 46]. Also, similar to findings by others[17, 53] we did not find the presence of AIDS diagnosis to be independently associated with MCS, which may further support the view that with time HIV-infected individuals may develop more effective coping strategies that could enhance their mental health[5, 22].

Mental comorbidity had a dramatic impact on mental functional health in our cohort (β: -6.25; 95% CL: -7.25, -5.25), which clearly shows the need for greater attention by both clinicians and policy makers in addressing mental health issues in this population of military personnel. The need for frequent and regular evaluation of the mental health of participants is further supported by the high prevalence of mental comorbidity in our cohort (over 25%). And as we stated earlier, depression was by far the most significant mental comorbidity in our cohort. There was no association between medical comorbidity and MCS. In our cohort, being African-American was positively associated with higher mental functional health which is similar to the findings in a non-HIV Military cohort which reported a higher MCS score among African-Americans compared to Caucasians[24]. While there may be need for further validation of this finding we are not sure if this has any clinical correlations, and is in fact below the 2 to 3 point difference in summary scores considered clinically meaningful[49]. Finally, our study showed no differences in MSC scores between NPI-HAART and PI-HAART (Tables 5 and 6) in both the unadjusted and adjusted models. Participants who were HAART-naïve or Off-HAART had statistically significant lower MCS scores compared to NPI-HHAART in the unadjusted model but not after adjustment (Table 5). On the other hand, participants on PI-HAART did not show any statistically significant differences in MCS scores compared to either the HAART-naive or Off-HAART groups even in the unadjusted model although that for the Off-HAART group was trending toward significance (p-value = 0.09).

Some of the limitations of our study include its cross-sectional nature, which may preclude conclusions on causality. Our study population was predominantly male (>90%) so generalizability to female should be applied cautiously. We also did not control for variables such as route of transmission as this was not captured at the time the surveys were administered due to the military “Don’t Ask, Don’t Tell” policy in place at the time[37]. It is worth noting that previous studies have, however, not found route of transmission to be independently associated with HRQOL[16, 17, 19, 40]. Finally, the use of the RAND SF-36 questionnaire, a generic HRQOL instrument, does not allow us to capture some important HIV-disease specific dimensions on quality of life such as cognitive functioning or sleep problems.

Our study had some major advantages. One, we simultaneously examined the differences in HRQOL measures in a large cohort of individuals on PI-HAART and NPI-HAART, as well as those who were HAART-naïve or Off-HAART. Because of the large sample size, we were able to adjust for many important confounding or mediating variables in our models. Our study also had a good representation of minority groups including African-Americans and Hispanics/other races. Also, the use of a norm-based generic HRQOL questionnaire (RAND SF-36) makes it easy for direct comparisons with different populations and settings including the general US population, non-HIV-infected US military population, other HIV cohorts as well as those of other chronic diseases that have used similar instruments. Finally, our models were fairly robust requiring removing only one influential observation from the PCS model and none from the MCS model.

## Conclusion

In conclusion, our study showed that modifiable risk factors associated with negative physical and mental functional health were mental comorbidity and CD4+ cell count <200 cells/mm^3^. Medical comorbidity and AIDS were only associated with poorer physical functional health. Addressing these risk factors may help improve the physical and mental functional health status of HIV-infected individuals in the United States’ Military.
